# Highly conductive electronics circuits from aerosol jet printed silver inks

**DOI:** 10.1038/s41598-021-97312-5

**Published:** 2021-09-13

**Authors:** Kacper Skarżyński, Jakub Krzemiński, Małgorzata Jakubowska, Marcin Słoma

**Affiliations:** 1 Micro- and Nanotechnology Division, Institute of Metrology and Biomedical Engineering, Faculty of Mechatronics, Warsaw University of Technology, 8 sw. A. Boboli st., 02-525 Warsaw, Poland; 2 CEZAMAT Centre for Advanced Materials and Technologies, Poleczki St. 19, 02-822 Warsaw, Poland

**Keywords:** Engineering, Materials science, Nanoscience and technology

## Abstract

Recently, low-cost electronics printed on lightweight, flexible and 3D shaped substrates are gaining importance in the markets of wearables and smart packaging. However, printed electronics do not meet the electrical performance of subtractive techniques because the resistivity of metallic printed patterns is still much higher than that of bulk material. To fulfil this need, low-resistive and easy printable inks for high resolution printed electronics techniques are needed. In this work, parameters of silver nanoparticles ink for micro-scale printed electronics technique, Aerosol Jet Printing, are being enhanced. To increase electrical conductivity and enhance printability, surfactants and dispersing agents were used to increase ultrasonic atomisation efficiency, obtain a uniform structure of printed lines, and narrow the width of printed patterns. Electrical measurements show a decrease in resistivity value in samples enhanced by cationic and non-ionic surfactants, by 95%, compared to initially prepared inks. Surfactant additions to silver nanoparticles Aerosol Jet Printing ink show promising features for application in modern electronics.

## Introduction

Recently in the electronics industry, especially in the areas of wearables and smart packaging, the need grows to produce low-cost electronics on lightweight, flexible, and 3D substrates^1^. In these areas of applications, high-resolution printing to ensure low dimensions of a product and high electrical conductivity for low energy consumption are crucial. To enable large scale production in such applications, printing techniques introducing high deposition speed are needed. That needs are met by direct writing techniques, allowing the contactless, additive and environmentally friendly, due to low material loss, printing of electronics. Among them, the technique combining high resolution and high printing speed with the uniform geometry and introducing the use of a wide variety of functional materials is Aerosol Jet Printing.

**Figure 1:**
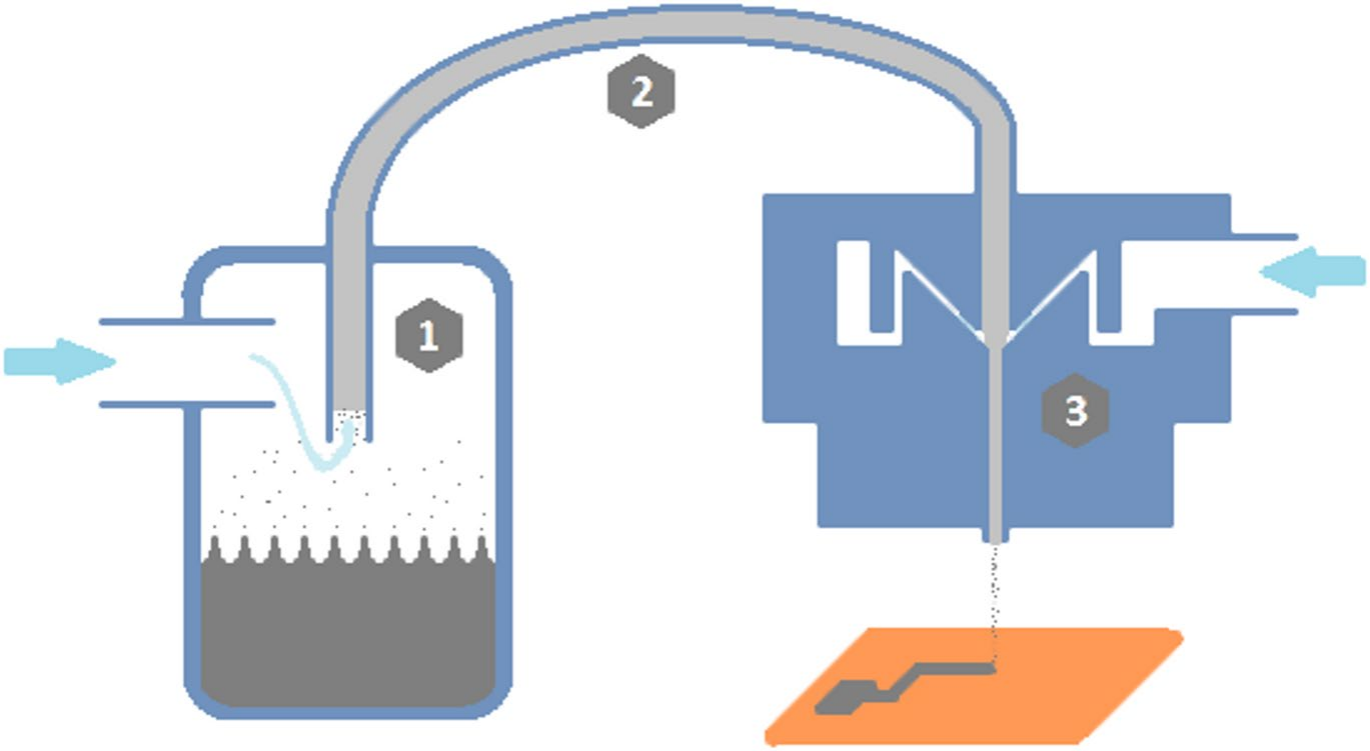
Aerosol Jet Printing schematics (1) ink atomisation process (2) Mist flow to deposition head caused by atomisation gas flow (3) Jet concentration by sheath gas and deposition on a substrate.

Aerosol Jet Printing (AJP), which schematic is shown in Fig. 1, is a printing technique that utilises a concentrated jet of atomised inks to create lines and patterns used as interconnects, passive and even active electronic components, e.g. top-gated field-effect thin-film transistor^2^. It is specially created for 3D substrates because created narrow aerosol jet ensures printing with nearly identical resolution in the range up to 10 mm from the tip of the nozzle to substrate^3^. This technique enables creating of interconnects of minimal width 8 µm and the same order of magnitude of electrical conductivity as for bulk silver^4^. It also shows promise for use in various applications, such as coplanar waveguides, antennas, detection electrodes, gates for displays, and collector lines for solar cells^5–8^. Due to the development of state of the art in this technique^3,9,10^, new process modelling approaches^11^ and its easy scalability, its application has started not only in research centres but also in commercial production.

Aerosol Jet Printing technique requires inks suitable to create a dense mist containing low diameter, homogenous droplets^12^ with high adhesion to the used substrate. Dense mist allows usage of a low total flow rate, which, with the proper sheath to mist ratio, reduces the path width dimension^13^. To achieve narrow lines with low resistivity, dedicated process parameters are crucial^12^, along with adjusting ink rheological properties.

**Figure 2:**
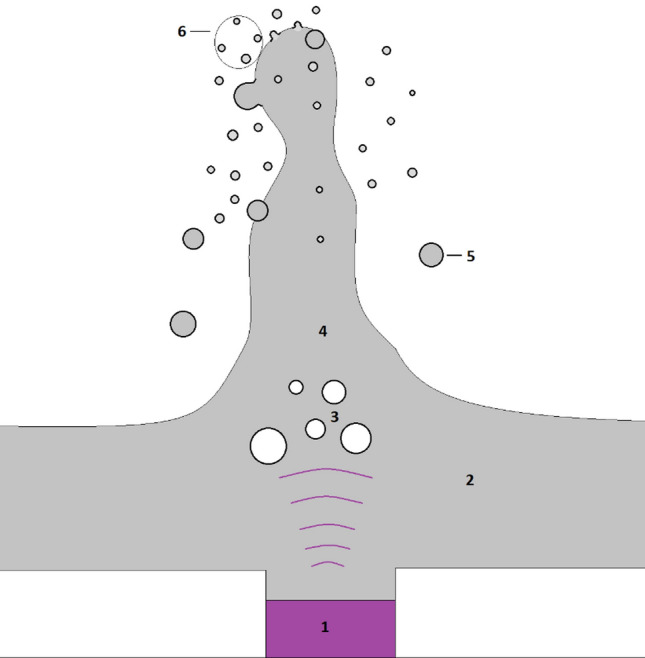
Ultrasonic atomisation schematics (1) Ultrasonic transducer (2) Liquid ink (3) Air bubbles (4) Standing wave (5) Big droplets falling back to container due to the gravitation (6) Aerosol mist.

In high-resolution applications, AJP utilises ultrasonic atomisation to create a dense aerosol mist. Schematics of the process is presented in Fig. 2. In this process, according to the cavitation-wave hypothesis by Eknadiosyants^14^, droplets formation is caused by a combination of instabilities in capillary standing waves on the liquid surface and acoustic cavitation bubbles beneath the surface of a liquid.

The value of the amplitude of the ultrasonic source that transfers ultrasonic energy to the ink needs to be above the threshold to break the liquid into droplets^15^. Even though ultrasonic atomisation does not support as wide variety of material as pneumatic atomization^16^ (ink viscosity need to be 0–20cP for ultrasonic and 0-1000cP for pneumatic), it creates a mist containing smaller droplets. To create the smallest possible droplets, crucial parameters need to be met, such as viscosity and surface tension of the ink, also crucial for atomisation efficiency (M. Renn, "Aerosol Jet Process Development Techniques", 2010). An increase in surface tension and shear viscosity not only increase the atomisation threshold, defined as acoustic intensity W·cm^-2^ at which atomisation occurs consistently within a 10 ms pulse but also increase the duration time of standing wave stability, understood by an experiment on viscous fluids. Tomotika^17^ proposed in that research the relation of these parameters in incompressible cylindrical jets as:

1 $$\tau \approx \frac{D\rho \eta }{\alpha }$$

where:

τ—duration time of jet stability.

*D*—jet diameter.

ρ—density,

η—coefficient of shear viscosity.

σ—surface tension of the liquid.

Inks for aerosol jet printing with ultrasonic atomisation contains three types of ingredients: solvent, functional phase and additives. The viscosity of the ink mostly depends on used solvents^18^, i.e. alcohols or deionised water, as the main liquid phase.

The functional phase needs to be small scale particles to allow the creation of solvent droplets containing functional particles. Since particles of functional phase with larger diameter contained in a solvent are heavy, they are more likely to fall back to the ink container than smaller ones. The best conductive materials for printed electronics are silver, gold and copper powders, due to their good conductivity, and vast availability of nanopowders with diameters below 0,2 μm. Also, carbon-based micro and nanomaterials, like graphene plates and carbon nanotubes, are suitable for these applications. Their concentration in ink must be chosen precisely to achieve a trade-off between atomisation related to the formation of a standing wave in low viscosity ink and a high concentration of conductive particles in ink. Additives are used to enhance ink properties, e.g. carbon nanotubes in silver ink are used to obtain higher conductivity^4^. To modify the surface tension of inks, enhance the dispersion of powders and inks stability over time, surfactants additions are used^19^.

Surfactants are chemical compounds with the ability to change the surface tension between gas, liquid and solid phases in mixtures^20^. Surface tension is measured as force per unit length tending to contract the surface, preventing small fractions of liquid molecules from vaporizing^21^. Surfactants are amphipathic molecules that consist of a hydrophilic head group structure attached to a hydrophobic hydrocarbon or fluorocarbon chain. The hydrophilic part can be anionic, cationic, amphoteric and non-ionic. In an aqueous solution, surfactants place themselves on the solution surface because the head is attracted to liquid molecules, and the chain is attracted to gas particles, lowering the surface tension^20^.

Surface Active Agents properties in liquid mediums were investigated in several publications. H. Chang-Jing and K.S. Lee found out that surfactants lower the surface tension^22^, which decrease the atomisation threshold. It also improved its adhesion to the substrate because of better wettability, which results in a wider contact area. C.T.Kosolia found out that surfactants improve particle dispersion^19^, which increase the percentage of ink droplets containing functional particles in the total number of droplets. On the other hand, Lu^23^ found out that surfactants prevent the coalescence of water droplets in aerosol mist, decreasing the diameter of the droplets in the mist. However, no detailed research for improving silver NP ink properties for the Aerosol Jet Printing technique by surfactants has been reported.

The conductivity/resistivity of several aerosol jet printed inks was evaluated in the literature. Commonly used commercial ink UTDAgX mean conductivity was measured by A.Mahajan^13^, with the results of 1.12·10^7^ S/m and E.Cantu^7^, with the results of 8·10^6^ S/m. Other most often used ink is Clariant Prelect TPS50G2, whose mean conductivity achieved by Ch. Oakley^24^ was 1.2·10^7^ S/m, and M.Morales—Rodrigues^25^ achieve even 2.38·10^7^ S/m. Several pieces of research modify commercial inks to improve their conductivity, and while solvent addition didn't lower the resistivity^26^ of ink sintered at low temperature (140–160 °C), SWCNT addition to ink sintered at temperature 350 °C lower the resistivity of the commercial ink^27^, achieving 2.8·10^−8^ Ωm. A novel alternative approach of reactive silver ink solution instead of nanoparticle-based results in even higher conductivity^4^ 4.6·10^−7^ S/m. However, the exact composition of commercial inks is a secret of its producer, and no detailed research for improving the conductivity of silver NP ink by surfactants have been reported.

This paper aims to develop ink with improving properties to print narrow lines with the highest conductivity. Several commercially available surfactants are evaluated, and the influence of surfactant concentration in silver NP ink on the conductivity of aerosol jest printed silver lines, sintered in a low-temperature process, is investigated. The conductivity enhancement will allow AJP to better meet the market requirements, resulting in a closer introduction of this technique for printed electronics applications.

## Methods

### Ink preparation

**Figure 3:**
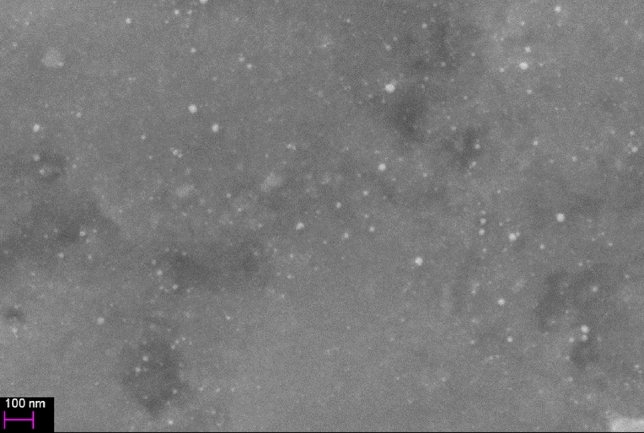
SEM Image of PAL silver nanoparticles, provided by the supplier. Observation has been made on scanning electron microscope LEO 1530.

The silver nanopowder (Silvercon nAg, Fig. 3) was mixed with surfactants and dispersed in toluene. The mixture was sonicated for 30 s four times, using 500 W Sonics vibracell 534 at 40% of the power. Then it was mechanically stirred for 5 min 2 times, using Retsch RM200. After each process, the loss of toluene was refilled with alcohol-based solvent to achieve 24 wt.% concentration of silver nanoparticles in ink. Solvents mixtures 1 and 2, used in the inks, are mixtures of organic solvents described in detail in previous work^28,29^.

To evaluate the influence of the surfactant concentration in ink on printing parameters, AKM-0531 (NOF Corporation) and solvent mixture 1 were used. Five different samples of basic silver ink (S0) and inks containing different concentrations of surfactant: 0.5 wt.% (S0.5), 1 wt.% (S1), 1.5 wt.% (S1.5), 2 wt.% (S2) were prepared.

In the evaluation of the influence of the type of surfactant used in ink on printing parameters, solvent mixture 2 was used. Four different samples, each containing 2 wt.% of surfactant, of Capstone (Sigma-Aldrich non-ionic surfactant) ink (CA), Triton X100 (Digma-Aldrich non-ionic surfactant) ink (TR), AKM-0531 (NOF Corporation Cationic surfactant) ink (D2), and Span 85 (Sigma-Aldrich nonionised surfactant) ink (SP) were created.

All of the ink samples are shown in Table 1.

**Table 1:** Ink Samples for surfactant concentration test (S0-S2) and surfactant type test (CA, TR, D2, SP).

Ink name	Solvent mixture type	Surfactant type	Surfactant concentration (%)
S0	1	AKM-0531	0
S0.5	1	AKM-0531	0.5
S1	1	AKM-0531	1
S1.5	1	AKM-0531	1.5
S2	1	AKM-0531	2
CA	2	Capstone FS-3100	2
TR	2	Triton X100	2
D2	2	AKM-0531	2
SP	2	Span 85	2

### Deposition process

**Figure 4:**
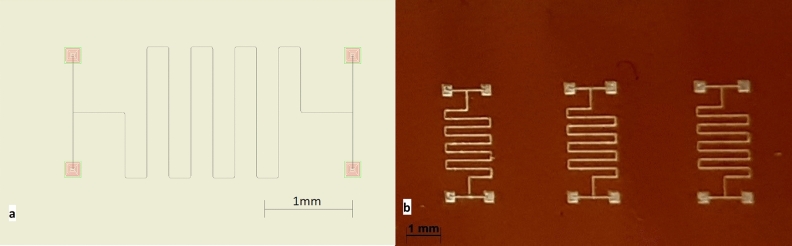
Pattern for four-point resistivity test (**a**) pattern design; (**b**) patterns printed on Kapton film and IR sintered.

The inks were atomised and printed with an M3D Optomecs Aerosol Jet Printing system and deposited at room temperature on Kapton HN film (DuPont, surface roughness in the same order as reported in other papers^30–32^). Air was used as the carrier gas. Figure 4 contains the standard pattern design for the four-point resistivity test. For each circuit, 4 layers were deposited with sheath gas flow 40 ± 5 sccm, carrier gas flow 26 ± 4sc cm, and printing speed 1.5 mm/min. All groups of samples were printed separately and sintered in the low-temperature photonic-sintering process^33^ by a 500 W halogen lamp at a 2 cm distance for 1 min^34^.

### Measurements

A four-point method for resistance measurements was used with Ketley 2001 multimeter. This method is used because of its precision in measurements of low resistances. The electrical resistivity was calculated from the equation:

2 $$\rho = \frac{R \cdot S}{L}$$

where:

ρ—electrical resistivity.

*S*—cross-sectional area of the printed line.

*L*—line length.

*R*—measured resistance.

The cross-sectional area used for the calculation of the electrical resistivity was used from the SEM observations on Auriga 60, calculated by multiplying line width and height with additional profile factor, also calculated from the SEM observations. Measurements of droplet diameter and minimum carrier gas flow that can carry mist droplets thru the aerosol system were made by deposition of aerosol droplets on filter paper with the minimum gas flow. Gas flow was measured by Bronkhorst EL_FLOW F-201CV-100-AGD-33-V flow controller, the presence of droplets marks was investigated by a 50-fold optical microscope, and their diameter was measured by VHX-900F Keyence microscope.

## Results and discussion

### Structure of printed lines

**Figure 5:**
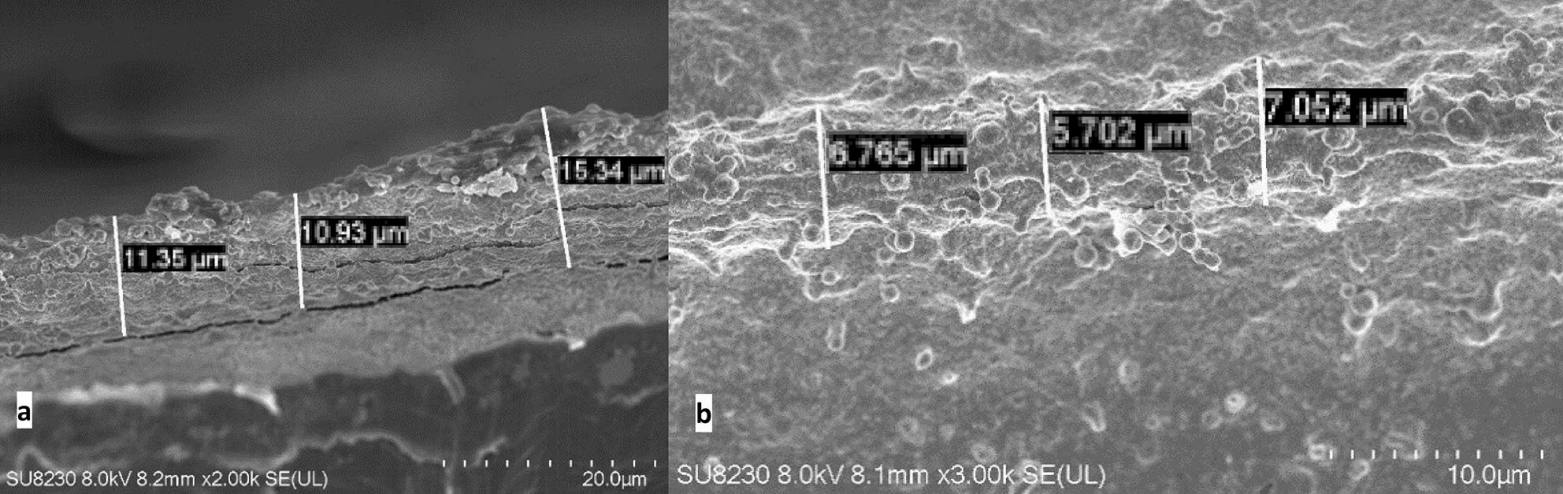
Measurements of the pattern, printed using S2 Ink, using Scanning Electron Microscopy images (Auriga 60) (**a**) the line width; (**b**) the line height.

**Figure 6:**
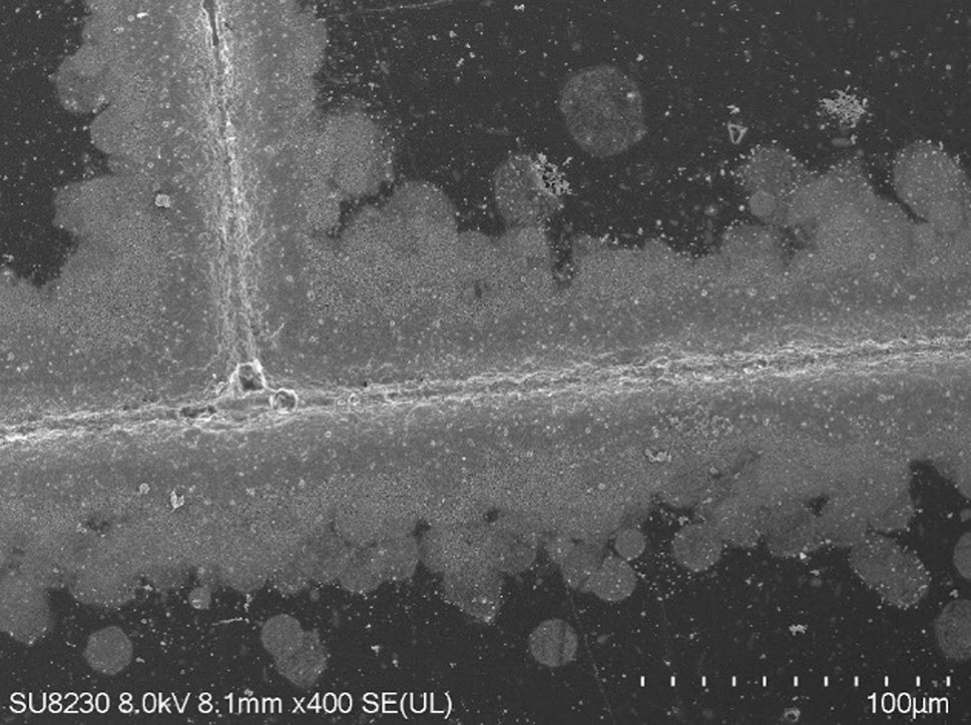
Overspray, characteristic Aerosol Jet Printing patterns element, emanating from the ink stream and expended by Saffman force^36^, visible for sample S2, in Scanning Electron Microscopy image (Auriga 60).

The morphology of the printed lines was investigated using Scanning Electron Microscope. In Fig. 5, the observation sample results for pattern printed using S2 test ink are presented. The measurements of mean line width differ from results acquire with an optical microscope because of the overspray shown in Fig. 6. The mean line width is 7.2 µm, and the line height is 9.8 µm (standard deviation 0.87 µm), and those parameters were used to calculate the resistivity of patterns.

**Figure 7:**
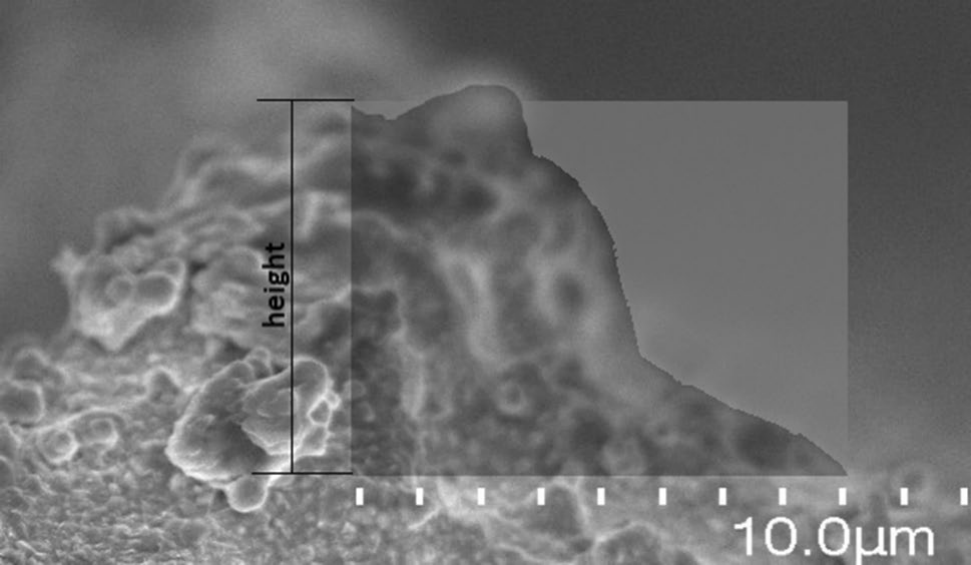
SEM micrograph of the cross-section profile obtained in the side view of the printed sample, with the additional image processing analysis used for the calculation of the effective profile of the printed path.

The effective cross-section area used in the calculations of the resistivity is estimated from the profile of the printed path, observed with the SEM microscope in the side view of the printed samples (Fig. 7). With the use of image editing software, we have calculated that the effective conductive area of the path is only 57% of the profile calculated by the multiplication of width and height of the layer, what is comparable to results accessed by other researchers^4^.

### Influence of Surfactants on the resistance of printed lines

Table 2 shows the mean resistance and cross-sectional area of all sample groups. Figure 7 shows the computed mean resistivity.

**Table 2:** Mean value of resistance and width acquire with an optical microscope of 8 printed 15 mm lines.

Ink name	Mean resistance (Ω)	Mean width (µm)	Mean droplet mark diameter (μm)	Minimum carrier gas flow (sccm)
S0	78.75	39.5	17.8	16
S0.5	51.4	30	19.2	16
S1	48.25	20.8	18.4	16
S1.5	63.9	17.5	17.5	16
S2	16.6	19.5	10.0	16
D2	18.3	35.4	8.0	20
SP	19.3	49.2	12.0	20
TR	50.1	50.2	50	0*
CA	> 1 M	–	16.5	20

*Atomisation was high enough to place droplets on the filter without any carrier gas flow.

**Figure 8:**
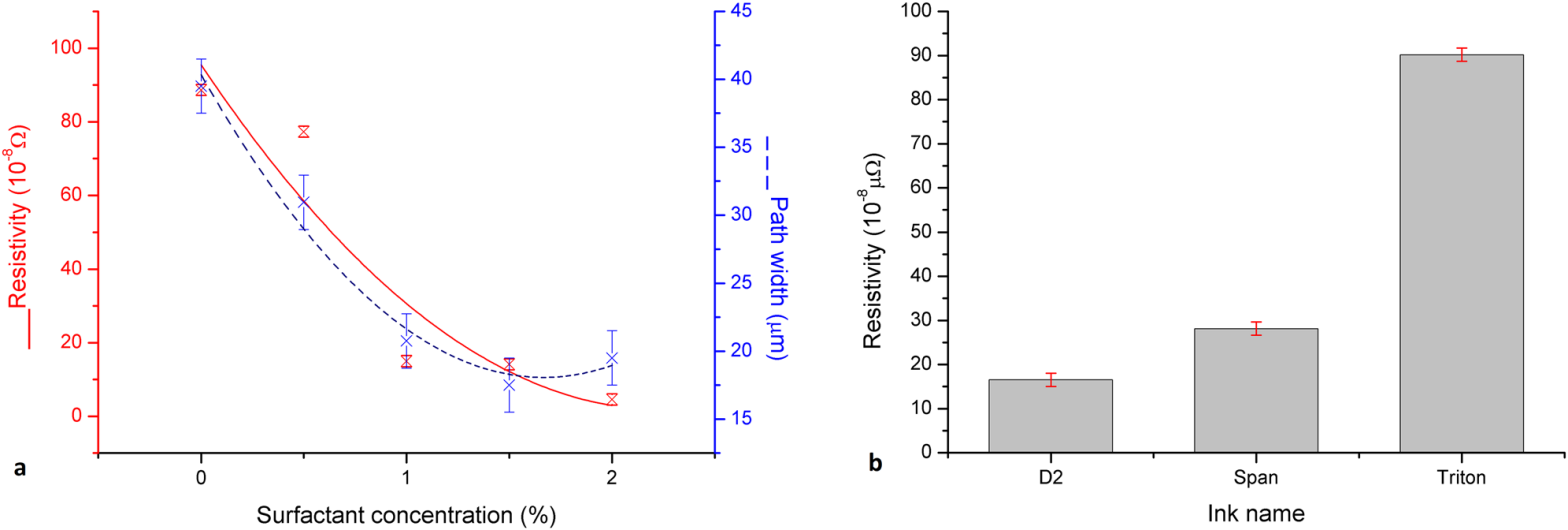
Charts of test results (**a**) computed mean resistivity and the path width for surfactant concentration test—Inks containing AKM-0531 as the surfactant in concentrations 0.5 wt.% (S0.5), 1 wt.% (S1), 1.5 wt.% (S1.5) and 2 wt.% (S2); (**b**) computed mean resistivity for surfactant type test—nks containing 2 wt.% of Capstone (CA)—not conductive, Triton X100 (TR), AKM-0531 (D2), and Span 85 (SP).

Surfactant concentration test shows that the addition of 2 wt.% AKM-0531 surfactant lowers the electrical resistivity by 95%. The addition of 0.5, 1 and 1.5 wt.% also improve resistivity, respectively. Figure 8 Shows that between the values 1.5 and 2 wt.% of surfactant addition, the measured mean width of the path increases. Because of this fact and the aim of this work to achieve narrow lines, higher surfactant concentrations will not be tested.

Surfactant type test shows that the electrical resistivity of printed lines containing AKM-0531 was 4 times lower than ones containing Triton X100. Lines printed using Span 85 has a higher resistivity than AKM-0531 lines but lower than Triton X100 ones, and Capstone lines were not conductive.

The result values calculated for D2 sample ink—resistivity 4.5·10^−8^ Ωm or conductivity 2.22·10^7^ S/m—are comparable to the best results for commercial inks, and five times better than achieved in research on modification of commercial ink in which sintering was conducted in comparable temperatures (2.3·10^ −7^ Ωm)^26^. A higher value of conductivity was achieved in research where authors used an alternative approach of reactive silver ink solution instead of nanoparticle-based (4.6·10^ −7^ S/m)^27^ and lower value of resistivity in research where the authors add carbon nanotubes to commercial ink and sinter them at temperature 350 °C (2.8·10^ −8^ Ωm)^4^.

Structural homogeneity of the printed lines, which can be enhanced with smaller droplets and lower gas flow, is the main factor that affects the electrical conductivity^4^. Droplet sizes and minimum gas flows were measured to investigate the results of conductivity measurements.

**Figure 9:**
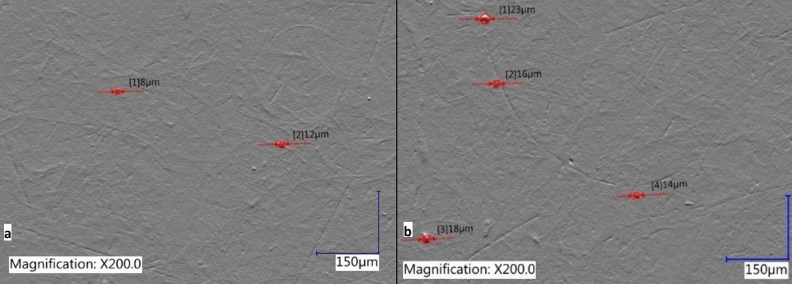
Example of measurements of droplet marks diameters for (**a**) S0 Sample; (**b**) S2 Sample.

Table 2 shows also measured droplet marks diameters (Fig. 9) and minimum carrier gas flow values. Mean droplet marks diameter decrease with the higher increase of the surfactant concentration. The lowest diameter was measured for the droplet marks from "Triton X100" ink.

Droplet mark diameters can be used for the comparison of different samples but not to directly determine the diameter of droplets. To investigate differences between marks on a paper filter and Kapton film, lines were printed on both of them using the same inks. This resulted in an 18% wider mean line width on the paper filter. We found that a decrease in the resistivity of printed lines is directly related to an increase in the surfactant concentration in used inks, thus decreasing droplets diameter. In Fig. 5, the silver pattern is porous and, therefore, can be considered air dispersed in silver. In that silver-air system, smaller droplets decrease the air amount. According to the resistivity-mixture rule, presented by Kasap^35^, if the dispersed phase is at least ten times more resistive with respect to the matrix, then:

3 $$\rho_{eff} = \rho_{c} \frac{{\left( {1 + \frac{1}{2}\chi_{d} } \right)}}{{\left( {1 - \chi_{d} } \right)}}$$

where:

$\rho_{eff}$—the resistivity of the entire material,

$\rho_{c}$—the resistivity of the continuous phase.

$\chi_{d}$—is the volume fraction of the dispersed phase.

For the S2 sample, which has the lowest resistivity, ${\chi }_{d}$- the volume of air in the porous pattern is calculated, taking into consideration ${\rho }_{eff}$ as the resistivity of the printed pattern, and ${\rho }_{c}$ as the resistivity of the bulk silver:

4 $$\chi_{d} = \frac{{\frac{{\rho_{eff} }}{{\rho_{c} }} - 1}}{{\frac{{\rho_{eff} }}{{\rho_{c} }} + \frac{1}{2}}} = \frac{{\frac{{4,5 \cdot 10^{ - 8 } \Omega m}}{{1,59 \cdot 10^{ - 8} \Omega m}} - 1}}{{\frac{{4,5 \cdot 10^{ - 8} \Omega m}}{{1,59 \cdot 10^{ - 8} \Omega m}} + \frac{1}{2}}} = 55\%$$

Triton ink results differ from the rest. That can be explained by the dependence of nozzle exit and optimum droplet diameters^36^. If droplets diameter is too low, line width spread significantly due to low Saffman force, which collimates bigger particles. Higher spread results in wider overspray and lesser homogeneity.

## Conclusions

In this paper, we present the preparation and printing of silver NP Inks with the addition of several types of surfactant (Triton X100, Capstone, Span 85, AKM-0531) with different concentrations—0, 0.5, 1, 1.5, 2 wt.%. Printed patterns were tested to define which surfactant concentration and type result in the lowest values of electrical resistivity. The best values of resistivity 4.5·10^−8^ Ωm were obtained for the S2 sample (ink containing the addition of 2 wt.% AKM-0531 and solvent mixture 1). The SEM observations allowed the measurements of the cross-section areas and revealed the presence of wide overspray, which occur due to smaller particle diameter caused by surfactants addition. However, it increases the density of ultrasonic mist and decreases the diameter of droplets, which results in more homogenous patterns. We prove that cationic and non-ionic surfactants, even in small concentrations, decrease the resistivity of printed patterns. This presents surfactants as promising additions to silver NP Aerosol Jet Printing ink for high conductive, flexible electronics.

## Data Availability

The datasets generated during and/or analysed during the current study are available from the corresponding author on reasonable request.
